# An Unusual Presentation of an Uncommon Drug: A Case Report on Phenibut Overdose

**DOI:** 10.7759/cureus.23913

**Published:** 2022-04-07

**Authors:** Christopher D Nedzlek, Anthony Michaelis

**Affiliations:** 1 Emergency Medicine, Henry Ford Health System, Wyandotte, USA

**Keywords:** nootropics, drug abuse, emergency medicine, overdose, phenibut

## Abstract

Phenibut (β-phenyl-γ-aminobutyric acid), a nootropic, gamma-aminobutyric acid receptor agonist, commercially marketed for anxiolysis, is increasingly recognized for its recreational abuse potential. We present the case of a 30-year-old-male with a history of polysubstance abuse, anxiety, and depression, who presented to the Emergency Department by ambulance for altered mental status with severe agitation, requiring both sedation and mechanical ventilation. It was later discovered that he had consumed phenibut, which he purchased through the internet. Currently, no readily available confirmatory laboratory tests exist to aid in the diagnosis of acute phenibut intoxication. We highlight the clinical distinctions between features of acute phenibut intoxication and withdrawal.

## Introduction

Phenibut (β-phenyl-γ-aminobutyric acid) is a gamma-aminobutyric acid (GABA) analog and agonist, acting with greater affinity for GABA-B than GABA-A receptors [1-3]. Phenibut is also known as phenigamma, Anvifen®, Fenibut®, and Noofen® [1,2]. It was created in the 1960s in the Soviet Union and was once used by cosmonauts for anxiolysis and cognitive enhancement [1,3]. Since that time, phenibut continues to be used clinically in Russia as an anxiolytic, antidepressant, nootropic (cognition enhancing), and mood-boosting agent [1-3]. Phenibut is known to have a wide volume of distribution and easily crosses the blood-brain barrier, allowing neurocognitive effects [1,3,4]. The effect of phenibut has been likened to that of baclofen and gabapentin, however with lesser potency [1,2,4,5]. Phenibut in the United States, while legal to possess, has not been studied or approved for licensing as a pharmaceutical drug [1,6]. Clinical use remains prevalent in Russia; however, the European Union and Australia have not licensed the drug for use [1,7]. Furthermore, phenibut fails to meet the definition of a dietary supplement in the United States by the Federal Drug Administration (FDA) [8]. Despite the lack of clinical use, recreational phenibut use continues to be reported in the United States and Europe [7].

Phenibut use typically exerts depressive symptoms on users, such as anxiolysis and sedation. With large consumption of GABA analogs like phenibut, there can be a paradoxical effect on anxiety [9]. Phenibut can also cause altered mentation, acute psychosis, delirium, reduced levels of consciousness, respiratory depression, tonic-clonic seizures, hypotension, temperature dysregulation, nausea, and vomiting [1-4,10].

## Case presentation

A 30-year-old male arrived to the Emergency Department (ED) by Emergency Medical Services (EMS). Initial presentation showed significant agitation, confusion, and resistance to care with frequent attempts to get out of the hospital bed. He had a Glasgow Coma Scale score of 13 with confused speech and was uncooperative with the staff. EMS providers reported that he was found by family and neighbors walking around his residence naked with incoherent speech. ED vitals included a heart rate of 159 beats per minute, a respiratory rate of 34 breaths per minute, a blood pressure of 164/89 mmHg, and an oxygen saturation of 96% on room air.

The physical examination showed a confused, tachycardic, tachypneic, and diaphoretic patient without signs of external trauma. He exhibited perseverating speech, answering all questions with his first name. His pupils were 3 mm and equally responsive to light. Neurologic examination exhibited normal reflexes and no clonus or focal neurologic deficit. His cardiopulmonary, abdominal, and musculoskeletal examinations were otherwise unremarkable. The patient remained agitated, was unable to cooperate with care, and subsequently required both chemical and physical restraints. Consecutive doses of 2 mg, 4 mg, 8 mg, and 16 mg of intravenous lorazepam within a 15-minute period were administered with minimal effect.

Review of prior hospital records revealed a prior medical history of anxiety, depression, and recurrent polysubstance abuse. The differential diagnosis was broad, including encephalopathy, acute drug intoxication or withdrawal, dehydration, metabolic derangements, and toxidromes. His presentation and history were concerning for sympathomimetic and anticholinergic toxidromes. Without additional historical clues or physical examination findings, the patient was intubated using rapid sequence intubation due to his inability to cooperate with care and to facilitate further testing and treatment.

Laboratory workup was significant for a leukocytosis of 13.1 x 10^9^/L without bandemia, a lactic acidosis of 5.9 mmol/L, an elevated anion gap of 18 with normal glucose. Complete blood count, comprehensive metabolic panel, arterial blood gas, and creatinine phosphokinase were all otherwise within normal limits. Ethanol, salicylate, acetaminophen, and ammonia levels were below detection. A urine drug screen was positive for benzodiazepines and cannabinoids. Blood cultures were obtained for possible bacteremia, later resulting in no growth. Additionally, a computed tomography (CT) scan of the head was obtained, which was negative for acute intracranial lesions and hemorrhages. A lumbar puncture was performed with unremarkable cerebrospinal fluid results.

After the initial workup was completed, the mother of the patient presented to the ED. The patient was last known normal three days prior and had reported his anxiety recently worsened. His mother was concerned he had ingested phenibut via a package he received through the mail two days prior. She showed the ED providers a 500-mg container labeled as “Phenibut” with Cyrillic writing, approximately half empty. The case was discussed with the local Poison Control Center and supportive care was recommended.

The patient remained intubated and sedated, and was given multiple liters of intravenous fluid for hydration. The patient was admitted to the intensive care unit (ICU) for supportive care. His ICU and hospital stay was uneventful, being extubated two days later. The Psychiatry service was consulted by the inpatient team due to recurrent polysubstance abuse history and concerns for an intentional overdose. The patient admitted to purchasing the phenibut from a “Russian website” because he “heard good things about it” and he “just wanted to get high” with a secondary goal of “improving my anxiety.” He reported heavy use of phenibut and acute ingestion on the day of his initial presentation while denying suicidal ideations. The patient was ultimately diagnosed with altered mental status secondary to acute ingestion and polysubstance abuse and was discharged home.

## Discussion

**Figure 1:**
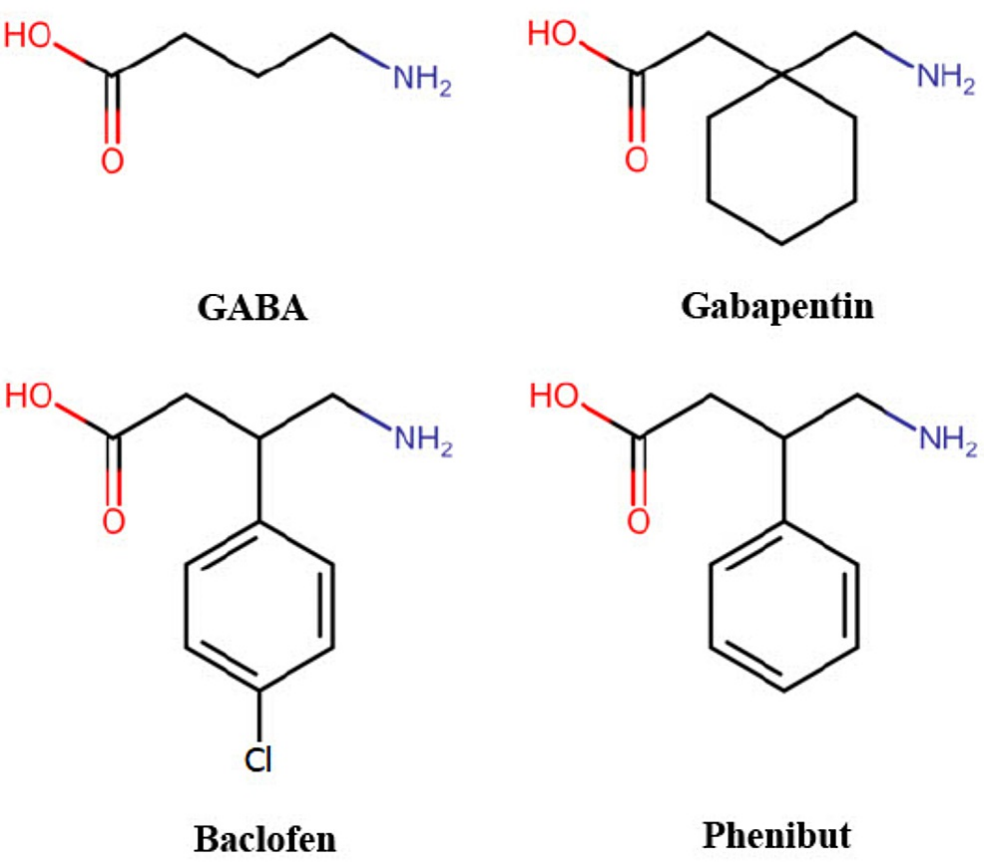
Chemical structure of GABA, gabapentin, baclofen, and phenibut GABA, gamma-aminobutyric acid

Phenibut is a GABA analog and exerts more effect on GABA-B receptors than GABA-A through neurotransmitter inhibition of voltage-gated calcium channels [3,4]. The phenibut molecular structure is comparable to baclofen and gabapentin (Figure 1). Acute use will stimulate the GABA receptors and cause sedating and central nervous system (CNS) depressive symptoms. Chronic use will lead to a downregulation of primarily GABA-B receptors and hyperactivity. Phenibut undergoes minimal hepatic metabolism and is excreted by the renal system unchanged [1].

Literature reviews of acute phenibut ingestion and acute withdrawal syndromes are limited. It is common for patients of both syndromes to have histories of depression, anxiety, insomnia, and polysubstance abuse [7]. Typically, patients with acute ingestion will have CNS depressive symptoms, while those with acute withdrawal will have excitatory symptoms. Phenibut withdrawal symptoms are similar to those of other GABA analogs such as baclofen, gamma-hydroxybutyrate, benzodiazepines, and alcohol [2-4,11]. This case report is unique as the patient exhibited both CNS depressive and excitatory symptoms. His presentation appeared like a GABA analog withdrawal, but his history was consistent with acute intoxication. We believe acute withdrawal was unlikely as the patient later admitted to recent recreational abuse preceding his hospitalization. It is unlikely he developed a significant level of tolerance with three days of use. His unusual presentation highlights the importance of obtaining accurate histories and considering both acute overdose and acute withdrawal syndromes.

Acute phenibut intoxication is typically showcased by CNS depressive effects. While phenibut intoxication lacks pathognomonic signs and symptoms, patients will likely have depressed mental statuses, stupor, or even unresponsiveness. Patients may report anxiolysis, euphoria, and hypnosis [7]. Other case reports have described patients having mildly increased anxiety, delirium, and psychomotor agitation [1,7]. Physical findings include acute dystonia, pupillary dilation, respiratory depression, and seizures. Hemodynamic instability is reported with tachycardia, hypotension or hypertension, and hypothermia [12,13]. Like other GABA analogs, there may also be a paradoxical effect (disinhibitory reactions) causing hyperactivity rather than depression [9]. We believe this to be the case for our patient. Treatment is aimed at supportive care with airway monitoring and respiratory support as needed, including mechanical ventilation and management of seizures [2]. No antidote or formal readily available diagnostic testing exists for phenibut intoxication [2]. Gas chromatography and mass spectrometry may be used for definitive identification, but offer little in time-sensitive, emergent situations [11].

Hyperactive signs and symptoms are more common with acute phenibut withdrawal syndrome, also known as phenibut abstinence syndrome. Acute withdrawal from phenibut may mimic serotonin syndrome, neuroleptic malignant syndrome, and benzodiazepine withdrawal [2,3]. Reported signs and symptoms of acute withdrawal include tremors, myalgias, nausea, and vomiting. Psychiatric signs and symptoms include increased anxiety, acute psychosis, delusions, hallucinations, disorganization, and increased depression. Neurologically, there may be decreased cognition, insomnia, psychomotor agitation, myoclonus, seizures, rigidity, and hyperreflexia. Patients may exhibit autonomic instability with fever and tachycardia. Just like its acute intoxication counterpart, treatment is aimed at supportive care. Literature shows that baclofen, phenobarbital, dexmedetomidine, benzodiazepines, olanzapine, and haloperidol have all been used to manage acute symptoms of phenibut ingestion [2-5,10].

## Conclusions

Altered mental status is a common ED presentation. We often consider the typical causes of altered mental status, such as infection, infarction, acute intracranial processes, endocrine disorders, and metabolic derangements, but emergency physicians must keep a broad differential diagnosis. As highlighted by this case report, we recommend considering emerging drugs of abuse and acute withdrawal as the etiology of a patient's altered mental status, especially in younger patient populations. With the increasing ease of access through the internet and social media, and lack of regulation within the United States, the potential for abuse with subsequent adverse reactions among patients is growing. Accurate histories are essential in making the correct diagnosis and treatment. This case highlights the need to consider phenibut overdose in any patient who presents to the ED with alteration in mental status or significant agitation.
